# Frequency of Hyperlactatemia in Intensive Care Patients within Tertiary Care Hospital in Pakistan

**DOI:** 10.7759/cureus.8264

**Published:** 2020-05-24

**Authors:** Taha Sheikh, Hina Shuja, Syed Hamza Bin Waqar

**Affiliations:** 1 Internal Medicine, University of Toledo College of Medicine and Life Sciences, Toledo, USA; 2 Internal Medicine, Abbasi Shaheed Hospital, Karachi, PAK; 3 Internal Medicine, State University of New York Downstate Health Sciences Center, New York, USA; 4 Internal Medicine, NYC Health + Hospitals/Kings County, New York City, USA; 5 Internal Medicine, Veterans Affairs Harbor Health Care, New York City, USA

**Keywords:** lactate, sepsis, intensive care units, hyperlactatemia

## Abstract

Objective

The aim of this study is to determine the frequency of hyperlactatemia in intensive care patients with sepsis in Civil Hospital Karachi.

Method

The cross-sectional study was conducted at a tertiary care hospital for six months and comprised all those participants who were older than 12 years of age, had sepsis, and were admitted in the intensive care unit (ICU) within 24 hours. Participants with suspected secondary acidemia from acute liver failure, end-stage renal disease, on anti-retroviral therapy, an overdose of salicylates, alcohols, acetaminophen, metformin user, intestinal resection, or bacterial overgrowth were excluded from the study.

Arterial blood samples were collected for lactate levels by the researcher himself, within 24 hours of admission to the ICU. The samples were stored in fluoride tubes and were kept at around four-degree centigrade temperature to avoid any alteration in lactate levels. Demographic details, diagnoses, vitals (blood pressure, heart rate, and respiratory rate) along with lactate levels and criteria for sepsis (including SIRS) of all patients were recorded in the proforma. Lactate level above 1.6 millimoles per liter (mmol/L) was taken as elevated (i.e. hyperlactatemia). SPSS version 16.0 was used for data analysis. Frequency and percentage were calculated for age categories, gender, and hyperlactatemia. Mean, and the standard deviation was calculated for the age of the patient and lactate levels. Effect modifiers were controlled through stratification for age, gender, co-morbidities, and duration of illness. p-value ≤ 0.05 was taken as significant.

Results

A total of 354 patients with sepsis were included in the study. The mean age of the patients was 42.93 (±19.03) years. Out of 354 patients, 169 (47.7%) were males, and 185 (52.3%) were females. Overall, the frequency of hyperlactatemia in intensive care patients with sepsis was 36.4%. The mean value of lactate was 1.963 mmol/L. With respect to stratification for age, hyperlactatemia was more frequent in the elderly patient population (47%) as compared to the young (31%) and middle (30%) aged patients with sepsis (p-value = 0.013). For gender, hyperlactatemia was more frequent in females (39%) than in males (33%). Concerning the duration of illness, hyperlactatemia was more frequent in those patients who were ill for more than one month (80%) than in those who were ill for less than seven days (30%) or 7 to 30-day period (36%) (p-value <0.001). Concerning co-morbidities, hypertension was the most common co-morbid condition in the study population (30%). Hyperlactatemia was significantly frequent in those patients who already had cardiac problem or stroke (66% [p value = 0.003] and 51% [p value = 0.013], respectively).

Conclusion

Hyperlactatemia is a common finding in patients with sepsis in an ICU. Being a marker of hypoxia, lactate level measurements can be of vital importance in a critical care setup, where they can be utilized to determine various factors such as mortality, morbidity, and duration of intensive care. Lactate levels thus should be evaluated in clinical studies, in correlation with various parameters more frequently. Lactate levels can also rise in various conditions apart from sepsis which merits further investigation.

## Introduction

Sepsis is the leading cause of mortality in the low-income countries of the world, according to the World Health Organization [1]. It is also a significant cause of morbidity and mortality in ICU worldwide [2-3]. About half of the patients admitted to intensive care units have an underlying infection, while hyperlactatemia (lactate level above 2.0 mmol/L) occurs in approximately one-third of patients admitted to the intensive care units [3-4]. Hyperlactatemia has been shown to correlate with the severity of illness, as a predictor of mortality in trauma and non-trauma patients, a screening tool for occult hypoperfusion in sepsis, and as a valuable end-point of early resuscitation in sepsis [5-7].

In critically ill patients, apart from sepsis, relative hyperlactatemia (lactate levels between 0.75-2.0 mmol/L) is independently associated with increased hospital mortality [7].

Since the inception of early goal-directed therapy by Rivers et al. in 2001, modified and improved by the Surviving Sepsis Campaign Guidelines, hyperlactatemia has been the starting point of the initial resuscitation bundle in severe sepsis and septic shock [8-9]. Furthermore, early lactate clearance time (that is the time needed to normalize lactate levels after high initial lactate levels) has been considered as even more reliable than single lactate measurement in predicting prognosis [10].

In a recent study by Jansen et al., early lactate-guided therapy signiﬁcantly reduced the ICU length of stay as well as ICU and hospital mortality, thus reducing costs of hospitalization and improving survival [11].

Thus, taking lactate as a reliable marker for global tissue hypoxia and microvascular dysfunction, the deteriorating condition of the patients can be identified in the earliest hours, even in the absence of hemodynamic instability. It is this “golden hour” when the resuscitation is most helpful. Thus, lactate levels not only help in important decision making in apparently stable patients but also serve as one of the reliable mortality predicting tools in septic shock patients and also help in reducing the financial burden on patients by reducing the length of stay in the ICU/hospital.

## Materials and methods

Study design and sampling

The cross-sectional study was conducted at a tertiary care hospital for six months duration. The study comprised all those who were admitted in the ICU of the hospital within 24 hours, were of the age 12 years or older, and had agreed to take part in the study. Informed consent was obtained after the participant was informed about the method and purpose of the study. The Ethical Committee approved the study of the hospital, and participants with acute liver failure, end-stage renal disease, receiving anti-retroviral therapy for AIDS, an overdose of salicylates, alcohols, acetaminophen, metformin use, intestinal resection or small intestinal bacterial overgrowth (promotes cellulose conversion into organic acids) were excluded from the study. The sampling technique used was non-probability convenience sampling. In a study by Khosravani H. *et al*., the incidence of hyperlactatemia in medical ICU patients was 36% in a cohort of 4935 patients [2]. Thus, keeping the level of significance at 5%, it was calculated that a sample size of at least 354 participants would be required with a 95% confidence interval and a 5% margin of error with the expected prevalence of hyperlactatemia being 36%.

Data collection

A structured proforma was designed and basic demographic details (name, age, gender and knowledge of any known co-morbidities), diagnoses, vitals (temperature, blood pressure, heart rate, and respiratory rate) along with lactate levels, and criteria for sepsis (including SIRS) of all patients were recorded in the proforma. Arterial blood samples were collected for lactate levels by the researcher himself, within 24 hours of admission to the ICU. To avoid any alteration in lactate levels, samples were stored in fluoride tubes and were kept at around four-degree centigrade temperature (in ice packs). The samples were immediately transported to the main laboratory of the tertiary University Hospital for immediate results. Samples were run on the Advia-1800 machine. Lactate level above 1.6 mmol/L was taken as elevated (i.e., hyperlactatemia); 1.6-1.4 mmol/L was taken as borderline high, while <1.4 mmol/L was accepted as normal lactate levels.

Data analysis

SPSS version 16.0 was used for data analysis. Frequency and percentage were calculated for age categories, gender, and hyperlactatemia. Mean, and standard deviation were calculated for the age of the patient and lactate levels. Effect modifiers were controlled through stratification for age, gender, co-morbidities, and duration of illness. P-value ≤0.05 was taken as significant.

## Results

**Figure 1:**
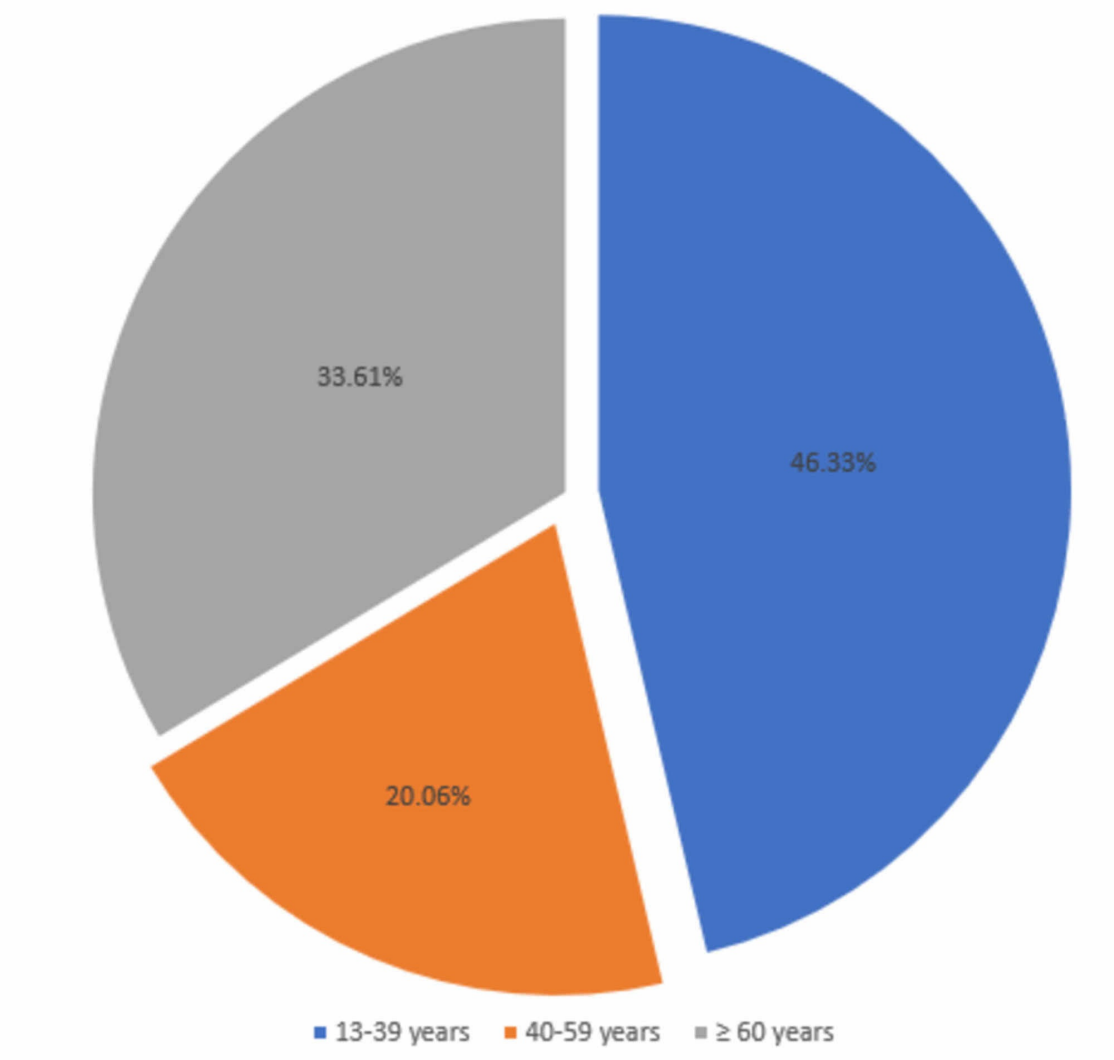
Distribution by age

A total of 354 patients with sepsis were included in the study. The mean age of the patients was 42.93 (±19.03) years (Figure 1).

**Figure 2:**
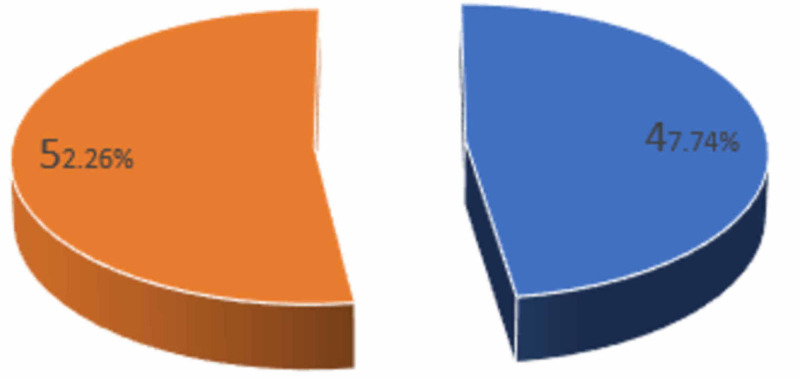
Distribution by gender Blue: males; Orange: females

Out of 354 patients, 169 (47.7%) were males and 185 (52.3%) were females (Figure 2).

Overall, the frequency of hyperlactatemia in intensive care patients with sepsis was 36.4% (Table 1).

**Table 1:** Frequency of cases with normal and elevated lactate levels (n = 354)

Lactate level	Cases	Frequency (%)
Normal	225	63.6
Elevated	129	36.4
Total	354	100.0

The mean value of lactate was 1.963 mmol/L. With respect to stratification for age, hyperlactatemia was more frequent in the elderly patient population (47%) as compared to the young (31%) and middle (30%) aged patients with sepsis (p-value = 0.013; Table 2).

**Table 2:** Age-wise distribution of normal and elevated lactate levels (n = 354)

Age Categories	Elevated Lactate	Normal Lactate	Total	P-value
13-39 years	51(31%)	113(69%)	164	0.013
40-59 years	22(30%)	49(70%)	71	
≥60 years	56(47%)	63(53%)	119	
Total	129	225	354	

With respect to gender, hyperlactatemia was more frequent in females (39%) than in males (33%; Table 3).

**Table 3:** Gender-wise distribution of normal and elevated lactate levels (n = 354)

Gender	Elevated Lactate	Normal Lactate	Total	P-value
<7 days	57	112	169	0.31
7–30 days	72	113	185	
>30 days	129	225	354	

With respect to duration of illness, hyperlactatemia was more frequent in those patients who were ill for more than one month (80%) than in those who were ill for either lesser than seven days (30%) or for 7-30 day period (36%) (p-value <0.001; Table 4).

**Table 4:** Distribution on the basis of duration of illness of normal versus elevated lactate levels (n = 354)

Duration of Illness	Elevated Lactate	Normal Lactate	Total	P-value
<7 days	65(30%)	155(70%)	220	<0.001
7–30 days	35(36%)	63(64%)	98	
>30 days	29(80%)	7(20%)	36	
Total	129	225	354	

**Figure 3:**
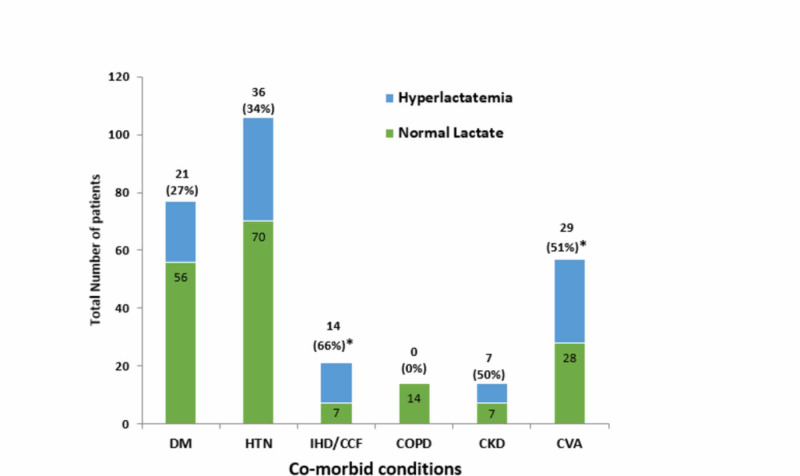
Frequency of elevated lactate level (hyperlactatemia) and normal lactate levels in various co-morbid conditions DM, diabetes mellitus; HTN, hypertension; IHD/CCF, ischemic heart disease and/or congestive heart failure; COPD, chronic obstructive pulmonary disease; CKD, chronic kidney disease; CVA, cerebrovascular accident *indicates significant p-value for the occurrence of hyperlactatemia in cardiac and stroke patients

With respect to co-morbidities, hypertension was the most common co-morbid condition in the study population (30%). Hyperlactatemia was significantly frequent in those patients with preexisting cardiovascular disease or stroke (66% [p value = 0.003] and 51% [p value = 0.013]), respectively (Figure 3).

## Discussion

Sepsis is defined as a systemic response to infection with changes in body temperature (above 38 degrees or below 36 degrees centigrade), heart rate (more than 90 beats per minute), respiratory rate (above 20 breaths per minute), and/or white blood cell count (above 12,000 or below 4,000/µL) [2]. Sepsis is a common and potentially life-threatening condition, especially in intensive care units. It is the single most common diagnosis in ICU and is the most common cause of multi-organ dysfunction and failure [2-3]. It is the leading cause of mortality in low-income countries throughout the world, and especially in intensive care patients [1]. 

Recently, the emphasis on the risk stratification of patients with sepsis is according to its impact on different body systems. Multiple scoring systems for organ dysfunction are in place, and many “markers” have been found [12-14]. Among these, lactate (or hyperlactatemia) is regarded as a marker of microcirculatory dysfunction, as it is a product of anaerobic metabolism at a cellular level [15].

Lactate levels depend upon the balance between lactate production due to global (shock, hypoxia), local (tissue ischemia), and cellular (mitochondrial dysfunction) factors on the one hand and lactate clearance depending on metabolic liver function on the other one [15]. Lactic acidosis is a major etiology of increased lactate levels. Further analyses reveal that decreased perfusion (type A) because of circulatory failure and hypoxia by respiratory failure are major anaerobic etiologies in acidosis. Other causes include underlying diseases (type B1), medications (type B2), and inborn errors of metabolism (type B3) in accordance with the Woods Classification of Lactic Acidosis [16].

Threshold lactate levels of above 1.6 mmol/L are considered hyperlactatemic as decided by the authors of this study. As lactate levels are closely correlated with hypoxia, lactate levels also determine the level of oxygenation. Thus, the main challenge was to compare lactate as a marker of tissue oxygenation determinant to other markers. Sepsis induces hypoxia through significant hemodynamic changes. This is reflected by parameters like the saturation of central mixed venous oxygen (MVO2), which determines hypoxia, as shunting due to sepsis leads to hypoxia and decreases oxygen pressures. However, it does not provide us a valid measuring point since it does not reflect the restoration of local tissue oxygen as in sepsis. Microcirculatory shunting can cause normal MVO2 despite the existence of severe local tissue hypoxia [17]. Thus, it is established that lactate levels are a superior marker of oxygen saturation [18-19].

We further took tonometry of the gastrointestinal tract as another comparative marker. In hypoxia, hyperacidity initiates, which is buffered by bicarbonate production, indicating carbon dioxide (CO_2_) levels. However, a study suggested that sublingual capnography was a better marker for CO_2 _concentrations [20]. Further tools such as orthogonal polarization spectral (OPS), Laser Doppler, and intravital microscopy (IVM) provide a more direct assessment of hemodynamic function [21].

Aerobic etiologies included cytokine-mediated glucose uptake, glycolysis, and mitochondrial dysfunction that explain the increased lactate production via lungs and WBCs in response to inflammatory stress, rather than tissue hypoxia. The other major etiology of hyperlactatemia is decreased clearance, as mentioned earlier. Impaired lactate clearance, rather than hypoxic tissue production of lactate, is the cause of hyperlactatemia in stable septic patients. Major clearance organs are liver and kidneys, and their dysfunction leads to hyperlactatemia as may be seen with increasing age, causing a decrease in kidney function physiologically, thus losing the reserve to endure the stress of sepsis [22-25]. This, in part, explains the occurrence of hyperlactatemia more in the elderly population (47%) in comparison to young (31%) and middle-aged (30%) patients as found by our data.

High lactate levels of above 2 mmol/L are associated with mortality. In the Surviving Sepsis Campaign Guidelines, the team of authors has pointed out that lactate levels of >4 mmol/L or hypotension (defined as systolic blood pressure of less than 90 mmHg) should immediately trigger the resuscitation of the patients according to the standardized protocol. Thus, in view of experts, the lactate level above 4 mmol/L is as important as hypotension, in the presence of sepsis [26]. This was further proved through pre-hospital measurement of lactate levels where, according to study, patients above 3.5 mmol/L exhibited greater mortality than patients with above 3.5 mmol/L. The study took vitals and Glasgow coma measurements but arrived at the conclusion that lactate and Glasgow coma scale were the most appropriate markers of in-hospital mortality. It is suggested that clinical intervention for lactate levels above four mmol/L should be frequently conducted for better management [27]. Further steps in the study reflected that decreased lactate levels decrease mortality [28]. Multivariate logistic regression demonstrated early lactate clearance had a significant inverse relationship with mortality (p=0.04). The investigators found an approximately 11% decrease likelihood of mortality for each 10% higher lactate clearance. Recent studies have shown that early lactate clearance is also associated with improvement in the biomarkers of inﬂammation and organ dysfunction [29]. We came up with finding that relative lactate level was also associated with mortality, as shown by a study by Nichol et al. [7].

In our study, hyperlactatemia was seen in 36.4% of the patients with sepsis. This finding is consistent with other international studies [4]. Although hyperlactatemia is also common after trauma, shock due to any cause, prolonged surgery, our study focused on sepsis because lactate levels are now included in management guidelines of severe sepsis. In the Surviving Sepsis Campaign and other descriptions of shock, lactate is regarded as a surrogate marker of the severity of microcirculatory dysfunction [9]. 

In our study, hyperlactatemia was more frequently seen in the elderly population (47% vs. 30% in younger patients) (p-value = 0.013). This finding reflects more severe derangement in this patient subset. This may be due to multiple causes, of which existing co-morbid conditions, late diagnosis of sepsis due to the absence of fever, and immune-compromised state due to advanced age, seem plausible. Thus, elevated lactate levels may trigger urgency in the resuscitation of these patients as well.

In our study, hyperlactatemia was more frequent in patients who were ill for more than a month (80%) against those who were ill for less than 30 days (36%) (p-value <0.001). This finding may reflect their advanced or complicated disease. Sepsis may be subacute too. Preence of hyperlactatemia signals complications in an on-going condition and necessitates immediate treatment.

Our study also showed that 57 (33.72%) of the male patients were hyperlactemic while 112 (66.28%) showed normal lactate levels. Similarly 72 (38.91%) females had hyperlactemia while 113 (61.09%) had lactate levels within normal limits. (p-value = 0.31; Chi Square value= 1.028).

Regarding co-morbid conditions, Hypertension was the most prevalent co-morbid condition among septic patients in ICU, constituting about one-third of the ICU admissions (34%, 70 normal lactate while 34 were hyperlactatemic). But the most significant finding in our study was the more frequent occurrence of hyperlactatemia in patients with Heart disease and CVA (stroke) (p-value <0.05). In septic patients with co-existent heart disease, there are chances of having both lactic acidosis type A (due to decreased perfusion or oxygenation) and Type B1 (due to underlying infection or inflammation) [16]. In patients with stroke, hyperlactatemia was explained by sepsis, secondary to urinary tract infection, and aspiration pneumonia in our study population.

In the MOSAICS (Management Of Severe sepsis in patients admitted to Asian Intensive Care Units) Study, researchers revealed that the frequency of lactate measurement was around 40 % in Asian hospitals. In this study, the compliance rates for the Surviving Sepsis Campaign guidelines’ resuscitation and management bundles were 7.6% and 3.5%, respectively [30]. This implies that there are no sufficient lactate levels measured neither any protocols followed to give lactate the significance it holds in hospitals of Pakistan and with the scope of this study in Karachi specifically. Thus research covers a vast scope of etiologies and association lactate levels with sepsis and its implication. This research is the only one of it's kind carried out in a tertiary care setup of Pakistan.

It is seen that there is a sense of complacency when any patient is diagnosed with sepsis or severe sepsis, as compared to the patient with myocardial infarction or stroke. Although rightly so, but prompt, effective, and guideline-based treatment of patients with sepsis and severe sepsis can also result in their better outcome, as shown by numerous studies on lactate [9,8,11].

This study has determined the frequency of hyperlactatemia in intensive care septic patients in a tertiary care hospital in Pakistan. This study may motivate the attending physicians, intensivists, and emergency care doctors, among others, to measure lactate in patients with severe sepsis, and to take hyperlactatemia as an important part of the protocol for the effective and evidence-based management and resuscitation of these patients, as recommended by the Surviving Sepsis Campaign guidelines.

Looking at the wealth of information that lactate measurement gives, as proven by plenty of studies, it seems practical to add lactate as a necessary tool in the management of patients with sepsis and severe sepsis.

## Conclusions

Hyperlactatemia is a common finding in patients with sepsis in intensive care units. As sepsis is the leading cause of morbidity and mortality in intensive care units throughout the world, any validated intervention, like lactate measurement, has a vast scope of application. The utility of lactate measurement and its implications on clinical decision-making in critically ill patients need further exploration. Specifically, the impact of hyperlactatemia on mortality, and other indices of morbidity (such as length of ICU stay) need to be explored. As lactate is elevated in many conditions, in addition to sepsis, further research is needed to enhance our understanding of the importance of hyperlactatemia in those conditions, as well.
